# OD33 Assessment Tool For MHealth Apps To Manage Depression (EvalDepApps Project)

**DOI:** 10.1017/S0266462324001648

**Published:** 2025-01-07

**Authors:** Carme Carrion, Meritxell Davins-Riu, Andrea Duarte-Díaz, Janaina Ferreira-Cavalcanti, Aïna Fuster-Casanovas, Estel Gelabert, Corpus Gómez-Calderón, Sònia Moretó-Melero, Antoni Pérez-Navarro, Noemí Robles, Josep Vidal-Alaball

## Abstract

**Introduction:**

The use of apps represents a revolution in mental health. Fast, versatile, and manageable, mHealth apps allow empowerment of patients and professionals and can even reduce stigmatization. There is not yet a standardized method to assess their effectiveness and safety. The objective of the EvalDepApps project is to develop an assessment tool for apps that have management of depression as the main goal.

**Methods:**

The EvalDepApps project follows several stages:

(i) Systematic review and meta-analysis following the PRISMA (Preferred Reporting Items for Systematic Reviews and Meta-Analyses) statement, to identify evidence about effectiveness and safety of mHealth interventions to manage depressive symptoms among adults, and criteria to be included in the app assessment tools. The primary outcome was the reduction of depressive symptoms, and only randomized controlled trials (RCTs) were included.

(ii) Delphi process with 30 participants (patients and healthcare professionals) to reach consensus about the criteria to be included in the tool.

(iii) Co-creation workshops with 12 healthcare professionals and 12 patients to co-design the EvalDepApps tool.

**Results:**

Twenty-nine RCTs were included. The most common elements were psychoeducation, goal setting, and gamification. Significant effect for mHealth interventions in reducing depressive symptoms compared with non-active control (95% confidence interval: −0.87, −0.37; I2=87%) was identified. Hybrid interventions combining mHealth with face-to-face sessions were the most effective. Any study-related adverse events were reported. Response rate was 59 percent (26/44) in round one and 52 percent (23/44) in round two. Twenty-eight out of 51 criteria (54.9%) were accepted by consensus. Proposals were received about the look and feel of the content, usability aspects, sections, and main features of the EvalDepApps tool.

**Conclusions:**

mHealth interventions, particularly hybrid ones, can be effective in reducing depressive symptoms. There is a need for personalized approaches. It is important to prioritize evidence-based principles and standardized evaluation tools. A set of 25 criteria will be included in the EvalDepApps tool that will be co-created thanks to the input given by healthcare professionals and people diagnosed with depression.

